# Assessment of the Osseointegration of Pure-Phase β-Tricalcium Phosphate (β-TCP) Ceramic Cylinder Implants in Critical Segmental Radial Bone Defects in Rabbits

**DOI:** 10.3390/vetsci12030200

**Published:** 2025-02-26

**Authors:** Daniel Cardoso Garcia, Larissa Eckmann Mingrone, Felipe Eduardo Pinotti, Leonardo Seade, Rosane de Melo, Ademar Benévolo Lugão, José Artur Brilhante Bezerra, Marcelo Jorge Cavalcanti de Sá

**Affiliations:** 1 Department of Surgery, Faculty of Veterinary Medicine, Federal University of Campina Grande (UFCG), Patos 58708-110, Brazil; danielcardosogarcia@gmail.com (D.C.G.); artur_brilhante@hotmail.com (J.A.B.B.); 2 Department of Surgery, Animal Care Barueri Veterinary Clinic, Barueri 06401-010, Brazil; laramingrone@gmail.com (L.E.M.); leoseade@hotmail.com (L.S.); rosanemelo31@gmail.com (R.d.M.); 3 Department of Surgery, School of Dentistry, São Paulo State University (UNESP), Araraquara 14801-130, Brazil; felipe.pinotti@unesp.br; 4 Biomaterials Laboratory, Institute for Energy and Nuclear Research, National Nuclear Energy Commission (IPEN/CNEN), São Paulo 05508-000, Brazil; ablugao@gmail.com

**Keywords:** biomaterial, bone critical defect, bone segmental defect, bone implant

## Abstract

Autografts, allografts, and synthetic bone substitutes are essential in reconstructive orthopedic surgery. Although autografts and allografts provide excellent skeletal integration, their use is limited by host morbidity and graft acquisition challenges. Synthetic materials like β-tricalcium phosphate (β-TCP) offer promising osseoconductive properties as a potential substitute. This study evaluated the osseointegration of β-TCP ceramic cylinder implants in bone defects in rabbits. Eighteen New Zealand rabbits underwent radial diaphysis ostectomy to create a critical segmental defect and were divided into three groups: Group A received β-TCP blocks, Group B received allogenous cortical bone grafts, and Group C underwent ostectomy without defect filling. Postoperative assessments included clinical evaluations, radiographs, micro-computed tomography, and histology at various time points to assess osseointegration and implant resorption. At the 120th postoperative day, Group B showed successful bone integration without infection. In contrast, Group A showed no osseointegration or resorption of the β-TCP implants, and Group C exhibited bone non-union. While β-TCP demonstrated biocompatibility, it lacked osseoconductivity, likely due to low porosity. β-TCP implants did not promote bone consolidation, suggesting that further research on porosity and shape is needed to improve their suitability for veterinary orthopedic reconstructive surgery.

## Simple Summary

Bone grafts are essential for repairing serious injuries, but traditional options like using a patient’s own bone or donated bone come with challenges, such as limited availability and potential complications. Thus, it is important to explore synthetic alternatives, like a biomaterial called beta-tricalcium phosphate (β-TCP), to substitute for these grafts. This study tested how well this material works by implanting it into rabbits with critical bone damage. The rabbits were divided into three groups: one received the β-TCP cylinder implant, another received donor bone, and the third had no treatment. Over time, the donor bone group successfully healed, while β-TCP did not integrate with the natural bone as expected. The untreated group showed no healing. Although the synthetic material was safe for the rabbits, it did not help the bone heal because it lacked the necessary structure to support new growth. These findings suggest that improvements in the implant’s design, like increasing its porosity, could make it more effective for bone repair. This research contributes to developing better options for treating bone injuries, which could benefit both human and veterinary medicine by providing safer, more accessible alternatives to traditional bone grafts.

## 1. Introduction

Tissue engineering is widely used to modulate the bone regeneration process and repair damaged bone tissue by promoting the proliferation and differentiation of bone cells within synthetic scaffolds, utilizing materials that substitute the lost or injured bone tissue [1,2]. Based on their sources, these materials used for bone defect reconstruction can be categorized into bone grafts (autogenous, allogeneic, or xenografts) and biomaterials, including non-degradable bone cement, metals, and ceramics [3,4].

A biomaterial is defined as any substance designed to interact with biological systems with the purpose of evaluating, treating, augmenting, or substituting any tissue, organ, or organism function [5,6]. Biomaterials used as bone substitutes should be structurally similar to bone regarding porosity and mechanical properties [7]. Furthermore, the material should facilitate good cell adhesion to its surface, be easy to use and handle, allow for easy sterilization, and be safe. It should also be free from antigenic, teratogenic, or carcinogenic effects and be cost-effective [2,8].

The promotion of osteogenesis, osseoinduction, osseoconduction, and osseointegration are key characteristics of an ideal bone graft material. Considering these properties, bone autografts remain the gold standard for the treatment of bone defects [7]. However, harvesting these grafts can prolong surgical time, increase risk of donor site morbidities, and present potential risks of infection or fracture during graft collection [3,9]. An alternative to autografts is the use of allografts, but this approach requires bone tissue banks, involves complex logistical procedures, incurs high costs associated with processing and preservation methods, and carries a risk of disease transmission [10,11].

Several studies on synthetic implants have been conducted to identify bone substitutes with properties that allow the effective stimulation, conduction, and integration of bone cells to promote bone healing [12]. Synthetic ceramic biomaterials, for example, can be produced with a composition similar to that of the inorganic bone matrix. These materials are not limited by availability and do not require additional surgical procedures for acquisition. However, their use is associated with certain disadvantages, including the absence of osteogenic or osseoinductive activity and poor mechanical performance under tensile stress due to their inherent fragility and high rigidity. Nonetheless, they exhibit excellent osteoconductive properties [13]. When ceramic is fixed to healthy bone, osteoid is directly deposited on its surface; subsequently, it is mineralized and remodeled as new bone [11]. Additionally, it is possible to enhance a ceramic osseoconductive matrix by incorporating bioactive agents that confer the additional properties necessary to replace autografts and allografts, such as bone marrow aspirate and bone morphogenetic proteins (BMPs) [3].

Among ceramic materials, tricalcium phosphate (TCP) is one of the most widely used, existing in two crystallographic forms: alpha (α-TCP) and beta (β-TCP). With the chemical formula Ca₃(PO_4_)_2_ and a Ca/P molar ratio of 1.5, TCP consists of approximately 39% calcium and 20% phosphorus. It is highly biocompatible and bioactive [12,14]. During the biological response to a bone defect, porous TCP is gradually resorbed at the implantation site, and new bone tissue forms within its structure. Porous biomaterials enhance the therapeutic efficacy of transplanted cells by reducing the risk of cell death [12,15,16]. Additionally, high-porosity, low-density composite ceramics provide an optimal surface area for neovascularization and bone regeneration [2,13].

The surface layers of TCP enhance its attachment to the adjacent host bone, stimulating osteoclastic resorption and promoting osteogenesis alongside implant resorption [13]. Moreover, TCP can be combined with other materials, such as hydroxyapatite (HA), autogenous bone, or BMPs, to improve its functionality and accelerate resorption. In its purest form, β-TCP exhibits superior osteoconductivity compared to α-TCP and HA [6,17,18]. However, one of its main disadvantages relative to HA is its limited structural support, attributed to its rapid resorption and high microporosity [3,19].

β-TCP implants are widely used in human medicine and dentistry as bone substitutes, primarily in granular form [3,12]. Therefore, this study aimed to evaluate the osseoinduction and osseointegration of pure-phase β-TCP cylindrical implants in critical segmental radial bone defects in rabbits using clinical, radiographic, microtomographic, and histological assessments.

## 2. Materials and Methods

The Bioethics Committee (CEUA) of the Federal University of Campina Grande (UFCG) approved the experimental protocols and the procedures used for animal care (Protocol number 046/2018). The procedures for the collection and storage of allogeneic cortical bone grafts, as well as the surgical and postoperative procedures, were performed at Animal Care Barueri Veterinary Clinic, located in the municipality of Barueri, state of São Paulo, Southeastern Brazil (23°30′41″ S, 46°52′36″ W). For this study, 19 male and female New Zealand rabbits, weighing between 3 and 4 kg were used. The animals were placed into 3 groups, consisting of 6 animals each. All animals were submitted to a radial diaphysis ostectomy to create a critical bone defect. In Group A, a synthetic implant of β-TCP was used to fill the bone defect. In Group B, a cortical bone allograft was used to fill the bone gap. In Group C, which was considered the control group of the study, the bone defects were not filled with any materials. The nineteenth animal was used for the aseptic collection of the two bones of the radius to make all the allogeneic cortical bone grafts that were part of the bone bank. These were later implanted in the segmental defects of the radius bones of the animals in Group B.

### 2.1. Implantation of β-TCP

The implants used in the experiment were made with a high degree of purity (99.9%) of ceramic composed of β-tricalcium phosphate (β-TCP) in its pure phase, without association with any other type of material. They were donated by the company Procell (Rio Claro, Brazil) and customized for this study, and thus were different from the commercial line already available. The implants were porous, custom-made cylindrical blocks that were 7 mm in length and 3 mm in diameter. The pore diameter of the implant was 1.1640 µm. These implants were already sterilized by gamma irradiation at the factory and packaged in ready-to-use surgical-grade paper packaging.

### 2.2. Acquisition of Allogeneic Cortical Grafts

Euthanasia was performed on one of the animals as described below (euthanasia section) for the aseptic collection of the forearm of both forelimbs. After obtaining the bone fragments for grafting, they were cleaned and washed through irrigation with 0.9% saline solution, and all tissue adhered to the cortical bone, including the periosteum, was removed. The medullary canals were also cleaned with the aid of a hypodermic needle, and any structures, such as blood vessels and bone marrow, were removed. Subsequently, the grafts, already completely free of any non-bone tissue, were washed again abundantly with 0.9% saline solution before being stored in a flask containing 98% glycerin solution, a product chosen to serve as a preservative for the grafts [20,21,22,23,24]. In this flask, the bone allografts were preserved for a minimum period of 30 days before being used.

### 2.3. Anesthetic Protocol

As a pre-anesthetic protocol, acepromazine (Acepran 0.2%, Univet S/A, São Paulo, Brazil) at a dose of 1 mg/kg and ketamine (Dopalen, Ceva Santé Animale, Paulínia, Brazil) at a dose of 40 mg/kg were used, both intramuscularly. After 15 min, the animals were catheterized in the marginal venous vessel of the ear and maintained in fluid therapy with Ringer’s lactate solution. Then, isoflurane (Isoforine, Cristália, Itapira, Brazil) was used for anesthetic induction by mask vaporization. For anesthetic maintenance, the same isoflurane was kept in an open anesthetic circuit using the mask mentioned above. The animals were monitored by electrocardiogram, heart rate, respiratory rate, blood pressure, oxygen saturation, and temperature [25,26].

### 2.4. Surgical Protocol

Before surgery, the hairs of the left forelimb of each animal were shaved and then disinfection was performed with skin cleansing with alcoholic chlorhexidine. A longitudinal cranial incision of approximately 6 cm was made in the skin over the topography of the left radius of each animal and the tissues adjacent to the bone diaphysis were dissected. In the middle third of the radial diaphysis, ostectomy was performed to remove a bone fragment of approximately 7 mm in length and create a critical segmental bone defect, according to Garcia et al. [27]. The measurement was marked on the bone with the aid of a Castroviejo compass and electrocautery. Then, the ostectomy procedure was performed with the aid of a mini high-speed electric drill (Dremel Bosch, BSH Store, Curitiba, Brazil) and a spherical dental drill (Broca Carbide FG n° 3, Angelus Prima Dental, Londrina, Brazil). Small holes were made transversely in the bone until it was possible to fracture it and completely remove the bone fragment.

Trimmings were performed on the remaining bone in order to make the bone defect as homogeneous as possible, with the osteotomies parallel to each other. Therefore, irrigation of the ostectomized sites was performed at the time of the cuts in order to avoid the occurrence of bone thermal necrosis. For the animals of Group A, the implants, which were ready for use, were removed from their sterilized casings and were placed in the bone defect created by the ostectomy. For the animals of Group B, cortical bone allografts were removed from the bone bank and placed in the bone defect created by the ostectomy. Both materials were inserted through pressure at the ostectomy site, so that their proximal and distal ends were in intimate contact and compressed with those of the animal’s bone.

At the time of using the allogeneic cortical bone grafts, they were removed from the 98% glycerin pot and subjected to an abundant prewash with 0.9% saline solution for 10 min before being placed in the bone defect to remove the glycerin and rehydrate the fragment to be used.

For the animals in Group C, the ostectomy site was left without any type of filling material, and the anatomical planes were sutured as described below.

Then, osteosynthesis was performed in Groups A and B to promote the stabilization of the operated limb and provide protection and mechanical support to the implants and grafts at the ostectomy site. For this purpose, 1.5 mm locked plates and screws made of titanium were used. Both the plates and screws used in this research were donated by the company Lincevet (Lincevet, Rio Claro, Brazil). Osteosynthesis was always performed in the form of a bridge over the implanted/grafted defect; that is, although the plate had 6 holes along its length, only 4 screws were used for blocking, 2 of which were placed in the two most proximal holes of the plate, and another 2 were placed in the two most distal holes, leaving the central part of the plate free of screws. After performing the ostectomy and osteosynthesis procedures, sutures were performed on the muscle planes, subcutaneous tissue, and skin with nylon 3.0 in a simple and conventionally separated pattern.

Care for the surgical wound stitches throughout the postoperative period, including dressing inspection, was performed twice a day. Stitches were removed after 2 weeks. To control the infection, enrofloxacin (Chemitril 2.5%, Chemitec, São Paulo, Brazil) was administered at a dose of 5 mg/kg, subcutaneously, once a day for 7 days. To control pain and inflammation, morphine (Dimorf, Cristália, São Paulo, Brazil) was used at a dose of 2.5 mg/kg, subcutaneously, in the immediate postoperative period, followed by tramadol hydrochloride (Tramadol, União Química Farmacêutica Nacional S/A, Pouso Alegre, MG, Brazil) at a dose of 5 mg/kg, subcutaneously, twice a day, for 7 days, in addition to meloxicam (Maxicam 0, 2%, Ourofino Saúde Animal, Cravinhos, Brazil) at a dose of 0.2 mg/kg, subcutaneously, once a day for 7 days [25].

### 2.5. Postoperative Evaluation

The animals from Groups A, B, and C were clinically and radiographically evaluated in the postoperative period (PO) for 120 days. The exact timing of each assessment is specifically described below for each study. Then, the animals were euthanized and µCT and histological studies were performed only for Groups A and B, for each bone segment containing an implant or bone graft, respectively.

#### 2.5.1. Postoperative Clinical Assessment

Daily inspections of the surgical wound and monitoring of the healing process were performed for the three groups. Cleaning of the skin and stitches was performed twice a day with 0.9% saline solution and 2% non-alcoholic chlorhexidine solution. The animals were also checked daily for swelling, redness, and edema at the operated site, as well as limb alignment, limb support on the ground, and gait status. This evaluation was purely macroscopic, subjective, and qualitative, through visualization and palpation of the operated site during the 120 days after surgery.

#### 2.5.2. Postoperative Radiographic Evaluation

All animals in the three groups were radiographically evaluated with digital radiographs postoperatively (System DR Wireless, Model Mars 1417V, TSI, iRay Technology, Shanghai, China; and Portable X Ray Model Orange 1060HF, Digicare, Oxson Technology, São Paulo, Brazil). Two radiographic views were performed on each animal, one craniocaudal (Cr-Ca), with the animal in the prone position, and another mediolateral (M-L), with the animal in the left lateral position. Five moments were recommended for the radiographs, called M0, M1, M2, M3, and M4. The referred moment M0 represented the immediate postoperative period, M1 was 30 days after the surgery, M2 was 60 days after the surgery, M3 was 90 days after the surgical procedure, and finally, M4 was 120 days after the surgical procedure.

### 2.6. Euthanasia

After a period of 120 days, each of the 18 animals used in the experiment was euthanized. For this, acepromazine was initially administered at a dose of 1 mg/kg intramuscularly, associated with ketamine at a dose of 40 mg/kg, also intramuscularly. Then, the marginal venous blood vessel of the rabbit’s ear was catheterized and sodium thiopentax (Thiopentax 1.0 g, Cristália, Itapira, Brazil) was administered at a dose of 30 mg/kg per intravenous route. After reaching an ideal anesthetic plane, 19.9% potassium chloride was administered at a dose of 1 mg/kg intravenously. Vital signs were evaluated with a multiparametric monitor and then checked by the veterinarian, who attested death due to cardiorespiratory arrest and absence of pulse [28].

### 2.7. Collection of Bone Fragments with β-TCP Implant or Bone Graft for µCT and Histology Studies

After euthanasia, and before the left radius of each operated animal from Group A and Group B was removed for µCT and histology, the regions where the β-TCP implants or allogeneic bone grafts were inserted were tested for possible contamination via culture tests for fungi and bacteria, as well as an antibiogram of the surgical site. The plates and screws were also removed, and two screws (second and third—most proximal and most distal, respectively) were sent for culture testing along with the previous swab.

With the aid of a Dremel and a dental drill, the bone was transversely sectioned in two places to obtain a smaller bone fragment (approximately 2 cm in length) containing the implant or graft. For this, only the implant or graft was left in the most central area and about 0.65 cm of the original bone of the host was left at both the proximal and distal ends. As a final piece for µCT examination and to make slides for the histological study, longitudinally, the new fragment was approximately 2 cm long and 3 mm in diameter. Again, the fragment was washed in 0.9% saline solution, packaged, and kept in a glass jar containing 10% formalin.

### 2.8. Evaluation by µCT

Micro-computed tomography was performed and evaluated at the micro-tomography laboratory of the Faculty of Dentistry, Universidade Estadual Paulista (UNESP, Araraquara, Brazil).

All samples from Groups A and B were processed and tomographically analyzed after the animals were euthanized. The collections of implanted bones and grafted bones were analyzed as reported by Irie et al. [29]. Bone fragments from the left radius of rabbits containing the β-TCP implant or an allogeneic cortical bone graft were removed from the flask with 10% formalin, where they were fixed for at least 48 h and subsequently stored in 70% alcohol.

Then, the samples were removed from 70% alcohol and supported on paper towels. They were wrapped in a paper towel and moistened with water using a syringe and needle. Then, the samples were stored in circular Styrofoam so that there was no interference in the generation of images and were inserted into the micro-tomography device.

The samples were scanned via micro-tomography (Skyscan, Aatselaar, Belgium) with the following parameters: camera pixel: 12.45; X-ray tube power: 65 kVP; X-ray intensity: 385 µA; integration time: 300 ms; filter: Al-1 mm; and voxel size: 18 µm^3^. The images were reconstructed, spatially repositioned, and analyzed with specific software (NRecon, Data Viewer, CTAnalyser, Aatselaar, Belgium).

On average, it took 17 min of scanning to generate the images for each part. All micro-tomographic procedures were performed by the same laboratory technician and interpreted by the same radiologist to avoid any bias in the technique and interpretation of the images.

### 2.9. Histological Evaluation

Histological evaluations were performed at the histological processing laboratory of the Faculty of Dentistry of São Paulo State University (UNESP, Araraquara, Brazil). All samples from Groups A and B were evaluated after euthanasia of the animals and after the acquisition of µCT images.

Randomly, for five samples from Group A and five samples from B, the slides for optical microscopy analysis were acquired through a grinding system (Exakt Apparatebeau, Hamburg, Germany), according to the protocol reported by Almeida et al. [17]. This methodology was used to analyze calcified bone samples.

For the other specimens of Groups A and B, the slides were subjected to histological study via optical microscopy using the hematoxylin and eosin staining methodology. In this case, the methodology was used for the analysis of decalcified bone samples. The decalcification process took place through the immersion and maintenance of bone pieces in EDTA. It took around 10 months to obtain a bone suitable for cutting with a conventional microtome. For the ceramic pieces, the biomaterial had to be removed to perform the cut, because even after this period, it had not decalcified as bone. As for the pieces with grafts, this was not necessary, as the graft decalcified exactly like the host bone, not preventing the cut by the microtome.

Slide images were obtained and evaluated using optical microscopy (Diastar, Leica, Wetzlar, Germany) at 2.5× magnification for calcified samples and 2.5×, 5×, 10×, 20×, and 40× magnification for decalcified samples.

All images were captured with a camera (Leica Application Suite V3.8; Leica, Wetzlar, Germany). For the 2.5× magnification images, three consecutive linear regions of interest (ROI) were identified. Photos of each ROI segment were taken (left side, center, and right side) and uploaded to a computer. With the aid of a computer program (Autostitch, UBC Industry Liason Office, Vancouver, Canada), the 3 parts of the photographs were reassembled to form 1 new complete photograph of the entire segment, which served for the histological analysis of the calcified bones.

As for the images of the decalcified bones, the above process was only necessary for the 2.5× magnification, not being necessary for the higher magnifications. For the magnifications of 5×, 10×, 20×, and 40×, we just took photographs of the slides directly. For magnifications greater than 2.5×, ROIs were identified in these same slides with a magnification of 2.5×. From these, images were made at the other aforementioned magnifications of the locations corresponding to the contact zone of the host bone with the materials that were used for filling and the adjacent areas.

## 3. Results

All animals from the three groups were clinically and radiographically evaluated over 120 days, except for one animal in Group B that died 76 days after the experiment due to an unknown cause.

### 3.1. Clinical Evaluation

Clinical evaluations were conducted daily during the first 15 postoperative days to assess pre-established parameters (Supplementary Material Table S1), and were subsequently performed on a weekly basis. None of the animals exhibited anatomical deviations of the bone axis, indicating good limb alignment. Limb support was observed within the first days of the postoperative period (PO), with no signs of lameness in the operated limbs, and gait was considered normal. Some animals experienced mild pain upon palpation of the ostectomy site and the plate, which was resolved over time through the use of analgesics and anti-inflammatory drugs.

None of the rabbits showed signs of swelling, hematoma, seroma, or edema in the operated limb. Some animals persistently removed their bandages, occasionally attempting to eat them, leading to the decision to leave the animals without bandages and perform only routine cleaning procedures. However, some animals bit and removed a few stitches from the surgical wound, but this did not result in skin damage or wound dehiscence. One animal in Group B developed a distal skin wound, away from the stitches, caused by self-trauma, which healed within a few days.

After the removal of stitches and complete skin healing, none of the animals exhibited any skin or subcutaneous tissue issues related to the presence of the plate, screws, implant, or graft. Additionally, no signs of infection or foreign body reaction, such as erythema, nodules, secretion, fistula, or pus, were observed in response to the synthetic implant or allogeneic cortical bone graft. Animals without filled defects from Group C also recovered well, showing no signs of infection.

An interesting observation in some rabbits was the persistent lack of hair growth or failure of regrowth on the shaved limbs after surgery. Not only did the radial region remain alopecic several months after the surgical procedure, but the shoulder of the same limb, the contralateral thoracic limb, and the ears, areas shaved for the surgical approach but not operated on, also exhibited alopecia.

**Figure 1:**
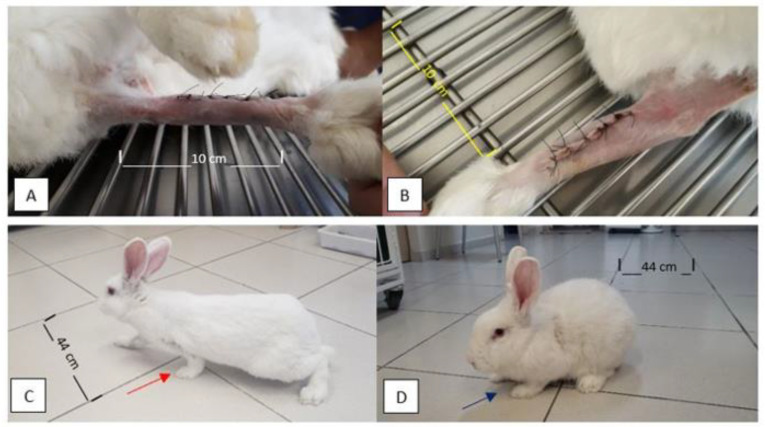
Photographic images of the limbs of the rabbits a few days after the immediate postoperative period and later at 120 days after the surgical procedure. Note the alignment of operated limbs and absence of edema, swelling, redness, signs of inflammation/infection, or foreign body reaction (**A**,**B**) at five days PO. In (**C**,**D**), note the good support of the thoracic limbs and load placement on the left thoracic limb at 120 days PO (red and purple arrows in (**C**) and (**D**), respectively).

During the 120-day postoperative evaluation period, the function, alignment, and support of the limbs remained satisfactory. There were no clinical signs of complications related to the surgical procedures, the ceramic implants, or the allogeneic cortical bone grafts used to fill the bone defects (Figure 1).

### 3.2. Radiographic Evaluation

All animals in Group B, where allogeneic cortical bone grafts were used, showed good progression and bone consolidation within 120 days. This included the animal from Group B that died before the stipulated evaluation period, at 76 days post-operation (PO). The animal had already exhibited bone neoformation and osseointegration between the graft and the host bone on the radiographs taken at M2 (60 days PO). Typically, radiographs from the immediate postoperative period (M0) and at 30 days PO (M1) still showed a proximal and distal radiolucent line between the bone edges and the graft. However, at subsequent time points, i.e., at M2, M3, and M4 (60, 90, and 120 days PO, respectively), these lines were no longer observed, indicating successful osseointegration between the bone and the graft. In some cases, bone remodeling was already evident by M4.

**Figure 2:**
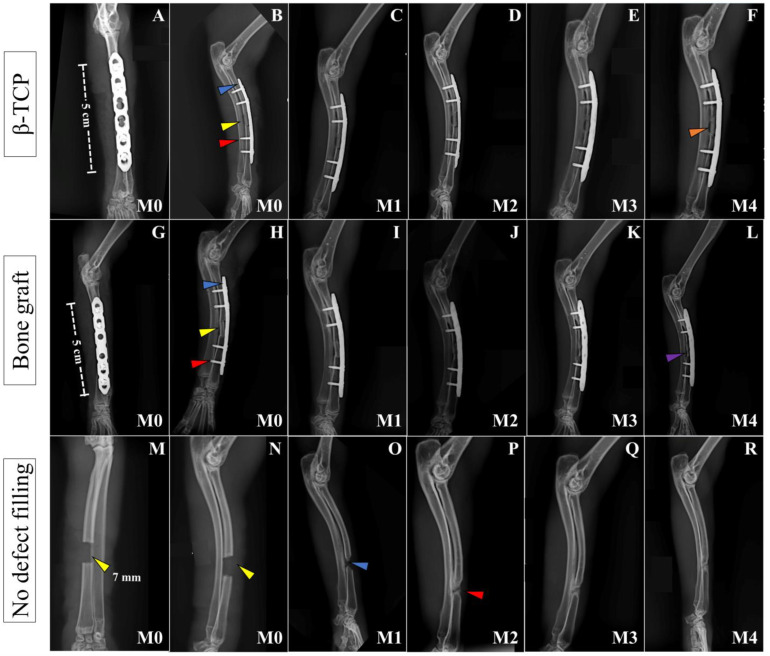
Postoperative radiographic images of the left forelimbs of animals in Groups A, B, and C, respectively, at moments M0 to M4. Craniocaudal images at immediate PO (M0) (**A**,**G**,**M**) and mid-lateral images at immediate PO (M0), and 30 (M1), 60 (M2), 90 (M3), and 120 days (M4) PO (**B**–**F**, **H**–**L**, **N**–**R**, respectively) of animals from Group A (β-TCP), Group B (Bone Graft), and Group C (no defect filling). In both groups with defect filling, note the ceramic implant and bone graft inserted at the ostectomy site (yellow arrow on (**B**,**H**)), the 1.5 mm titanium plate (blue arrow in (**B**,**H**)), and the 1.5 mm titanium screws (red arrow in (**B**,**H**)). In (**F**), at M5, the integration of the implant and bone cannot be confirmed (solid orange arrow) because of the radiotransparent lines between the bone and the implant, despite its perfect positioning. In (**L**), at M4, it is possible to notice the integration between the bone and the graft, with the beginning of bone remodeling at the bone critical defect site (solid purple arrow). In (**M**,**N**), at M0, we can see the ostectomy site, which is 7 mm in length (yellow arrows). In (**O**), at M1, there is initial growth of bone inside the defect (blue arrow) that increases until (**P**) at M2 (red arrow). From that moment, it seems that the bone growing process stops, since the image inside the defect is the same for the next 60 days (**P**–**R**) from M2 to M4.

In Group A, some images suggested possible biological activity between the implant and the host bone, with no signs of bone or implant resorption observed at M4. Radiolucent lines were still present between the implant and the bone edges, indicating that complete osseointegration had likely not occurred. In Group C, at 120 days post-operation, there was no evidence of complete bone growth or integration between the proximal and distal bone edges, resulting in bone non-union in all animals in the group (Figure 2).

In all cases where osteosynthesis was performed, no fractures, loosening of bone plates or screws, or signs of osteomyelitis were observed. On the contrary, good alignment of the limb, as well as proper positioning of the plates, grafts, and implants, was consistently noted. In one animal from Group B, only three screws were inserted into the plate instead of the four recommended for the experiment due to an iatrogenic fracture of the bone during drilling for the third hole (from proximal to distal), which did not allow the drilling and insertion of a fourth screw.

### 3.3. Evaluation of the Surgical Procedure

**Figure 3:**
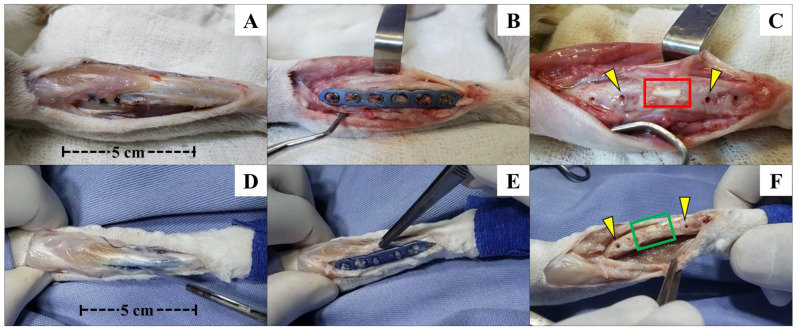
Photographic images of the left forelimb of an animal from Group A (β-TCP; (**A**–**C**)) and another from Group B (allogenic cortical bone graft; (**D**–**F**)) shortly after euthanasia, with exposure of the operated site for removal of bone fragments for µCT and histological studies. Photographic images showing good limb alignment and no signs of inflammation, infection, or foreign body reactions (**A**–**F**). After removing the plate and the screw, it was possible to notice the good alignment of the β-TCP implant (**C**) and allogenous cortical bone graft (**F**) in relation to the bone and the possible osseointegration of the implant with the surrounding tissues inside the bone defect and the bone edges. Macroscopically, it seems that there was no implant resorption. The yellow arrows in (**C**,**F**) show the positions of the second and third screws (left to right direction), corresponding to the proximal–distal direction of the forelimb. The red and green squares indicate the positions where the β-TCP implant and allogenous graft were inserted in the bone defect. In Group B, osteointegration seems to be more effective and macroscopically visible than Group A. For both groups, it is visible that there was not any kind of foreign bone reaction or infection in the bone or other tissue surfaces below the metallic implants, as seen after plate and screw removal.

From a surgical perspective, as observed 120 days after surgery during euthanasia (and following the death of the animal in Group B at 60 days post-operation [PO]), all twelve animals in Groups A and B showed the plates and screws to be well positioned and aligned with the bone, with no alterations over the evaluated period. Connective tissue growth was noted on the superficial bone lamina and plate, along with exuberant mineralized tissue growth around and over the plate in some areas in both Groups A and B (Figure 3).

There were no signs of infection at the operated sites. Upon removal of the plates and screws, it was observed in all rabbits from Groups A and B that the implants and grafts were well inserted and properly aligned at the ostectomy site. Macroscopic examination suggested possible integration between the recipient bone and the materials inserted into the bone defect, as there was no movement observed, even when pressure was applied with the fingers. The implants and grafts remained firmly in position.

No signs of bone resorption or any indications of loss of biological activity at the ostectomy site were observed, whether due to the ostectomy itself, performed with a dental drill, or the presence of synthetic biomaterial or bone graft preserved in 98% glycerin. At this point, culture and antibiogram tests were conducted, all of which yielded negative results.

After disarticulation of the limb and the removal of all soft tissue adhered to the bone and implant, a significant amount of connective tissue was observed surrounding the implant and graft, as well as at the interface between the implant or graft and the proximal and distal edges of the ostectomy site.

After the careful removal of this tissue, the interaction between the biomaterial, the bone graft, and the host bone was evident, with no signs of mobility or loosening. The implants remained intact, showing no signs of resorption, while the allogeneic cortical bone grafts appeared fully integrated with the host bone, indistinguishable from the recipient bone in rabbits after 120 days. Even during the cutting of the bone to obtain the 2 cm fragment for histological slide preparation, both the β-TCP implant and the allogeneic cortical bone graft remained stable, showing no signs of destabilization during manipulation of the fragment.

### 3.4. Micro-Computed Tomography and Histological Evaluations

**Figure 4:**
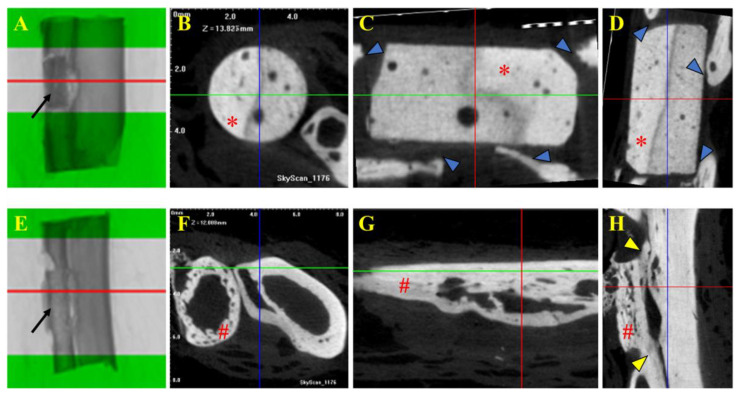
Tomographic images of selected fragments of animals from Group A (β-TCP; (**A**–**D**)) and Group B (cortical allogeneic bone graft; (**E**–**H**)). Tomographic images of 2 cm bone with β-TCP implant (Group A) and bone graft (Group B). (**A**,**E**) Middle part of the implant and graft as region of interest (ROI) for μCT analysis and acquisition of cross-sectional images. Note the implant and bone graft inserted in the receptor bones (black arrows in (**A**) and (**E**), respectively). (**B**,**F**) Trans-axial cut. (**C**,**G**) Coronal cut. (**D**,**H**) Sagital cut. * β-TCP implant. # Allogenous cortical bone graft. Note that there is no implant–bone osteointegration seen in μCT images of Group A (blue arrows in (**C**,**D**)), but on the contrary, there is positive osteointegration between the bone graft and bone, as shown in (**H**) (yellow arrows).

In the μCT images, no osseointegration was observed between the β-TCP implant and the bone in the animals from Group A. In contrast, visible osseointegration between the allogeneic cortical bone graft and the bone was evident in the animals from Group B. In Group A, the implants were well positioned with no signs of mobility at their initial insertion sites. Although the edges of the recipient bone grew towards the implant, they did not integrate with the ceramic to form a single, continuous structure, as seen in the images. The cylindrical blocks of the implants retained their original size, and no clear signs of resorption were observed. However, some small radiolucent circular dots were noted inside the implant, contrasting with the rest of the structure. These could potentially represent the early stages of implant resorption or areas of greater porosity within the material (Figure 4).

In Group B, the bone grafts exhibited evidence of osseointegration, as well as the early stages of bone remodeling. The edges of the recipient bone grew towards the graft, eventually connecting and forming a single, continuous structure, visible in the images. The grafts were well positioned and showed no signs of mobility. There also appeared to be integration with the adjacent bone, the ulna, where the two structures could be observed in direct contact (Figure 4).

**Figure 5:**
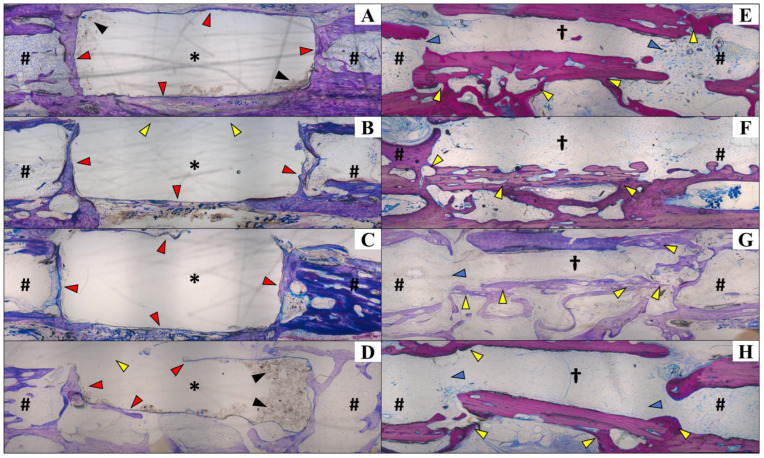
Histological analysis of calcified sections to visualize the positions of the β-TCP implant (Group A; (**A**–**D**)) and bone graft (Group B; (**E**–**H**)), as well as their interfaces with the receptor bone. Staining was performed using Exakt protocol. In Group A, the β-TCP implant reacted with the resin during the fixation protocol, leading to its dissolution (**A**–**D**), except for some remaining intact portions (black arrows in (**A**,**D**)). Notably, no newly formed bone was observed within the implant area. The receptor bone edges remained intact, encompassing the implant (red arrows in (**A**,**C**)), except in certain areas (yellow arrows in (**B**,**D**)) where minor resorption was observed. There were no signs of foreign body reactions, indicating the implant’s biocompatibility. The implant appeared to retain its original shape 120 days post-implantation, showing no evidence of resorption. In Group B (**E**–**H**), the allogenous bone graft successfully integrated with the receptor bone, forming direct connections and undergoing remodeling. The integration was evident along the graft edges and surfaces (yellow arrows in (**E**–**H**)). In most cases, the graft and receptor bone communicated through a medullary channel (blue arrows). Center of the implant (*); proximal and distal portions of the bone (#); center of the allogenous cortical bone graft (†); ×40 magnification. Histological slides with 40 µm thickness.

After acquiring the μCT images, the 2 cm bone samples from Group A were prepared for histological analysis. Five of the six calcified bone samples were randomly selected for the Exakt protocol. Unexpectedly, the β-TCP implants reacted with the resin or alcohol baths during the fixation protocol and dissolved for unknown reasons, with only some parts remaining intact after the procedure. No newly formed bone was observed within the area corresponding to the implant. However, the bone edges did not resorb, and connective tissue was observed surrounding the implant, except in some areas at the most cranial part of the implant, where no tissue was present. The bone edges formed a sort of barrier between the implant and the surrounding tissue. There were no signs of a foreign body reaction, indicating biocompatibility. The ceramic implant retained its original shape, with no signs of resorption, even after 120 days (Figure 5).

The allogeneic cortical bone graft formed connections with the recipient bones and remodeled in most cases, showing integration between the graft’s edges and surfaces. In most cases, the graft and bone communicated through a spinal canal, as if they were a single structure. The osseointegration process was more efficient than in Group A. Additionally, greater bone neoformation activity was observed in Group B compared to the other groups. As in Group A, there was no evidence of a foreign body reaction in Group B (Figure 5).

The other two samples (one with β-TCP and the other with bone graft), which were randomly selected for histological study using the hematoxylin and eosin staining process, underwent a bone decalcification procedure to prepare the slides. Regarding the synthetic ceramic implant from Group A, its position within the bone defect was much clearer to observe compared to the graft from Group B. This was because the implant’s color was much whiter than the rabbit’s bone, making it more noticeable macroscopically. Additionally, the implant’s surface had a rounded appearance, in contrast to the original bone of the rabbit, whereas the graft exhibited a flatter surface relative to the synthetic implant.

The decalcification process took about 10 months, and for the β-TCP pieces (Group A), it was necessary to remove the ceramics that were inserted into the bone, as they did not decalcify like the adjacent host bone and retained their initial hardness.

For Group B, the decalcification process was performed without complications. After the procedure, a longitudinal cut was made in the specimen, and it was practically impossible to identify the region where the bone graft had been implanted. The only color differences macroscopically visible in the specimen were a darker, well-defined area corresponding to the screw insertion site and a slightly lighter color of the graft compared to the bone.

**Figure 6:**
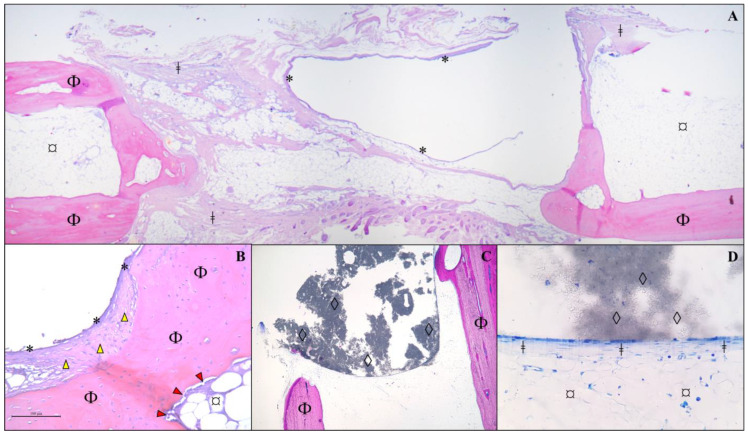
Histological analysis of a section of bone fragment/implant from an animal from Group A, 120 days after receiving a β-TCP cylinder implant. Protocol using hematoxylin and eosin in a decalcified bone fragment. (**A**) Observe the cavitation lined by a delicate fibrous capsule (*) and surrounded by a thin deposition of fibrous connective tissue (ǂ) between the bone lamellae of the host mature bone (Φ) and adipose tissue (¤). (**B**) At higher magnification (×10), thickening of the capsule (*) is noted, characterized by fibrocytes (yellow arrowhead) and an extracellular matrix (mature fibrous connective tissue/fibrosis) closely associated with the bone matrix (Φ). Additionally, an immature fibrous matrix (red arrowhead) is observed lining the bone lamella (Φ) in contact with extramedullary adipose tissue (¤). (**C**) Presence of amorphous granular black material (β-TCP implant remnant/◊) interspersed among bone lamellae (Φ) in direct contact with the implant (×5 magnification). (**D**) At higher magnification (×40), a thin layer of connective tissue is observed in direct contact with the implant (◊), surrounded by extramedullary adipose tissue (¤). No newly formed bone cells are present.

In the histological section of decalcified bone from the animal in Group A, it was observed that there was no invasion of bone cells into the area of the implant, nor any formation of neovascularization. The edges in contact with the implant showed only fibrous tissue and connective tissue growth, with no osseointegration of the β-TCP implant to the host bone. Fibrocytes and fibroblasts were present at the implant margins, but osteoblasts and osteocytes were only found in the lamellar bone area corresponding to the host bone, not in the expected bone neoformation associated with implant bioactivity. The medullary canal was closed with a bone “wall”, but there was no invasion of this bone to connect with the implant. However, there was no resorption of the bone ends. In a deeper-cut area, some remnants of the ceramic material were still visible (Figure 6).

**Figure 7:**
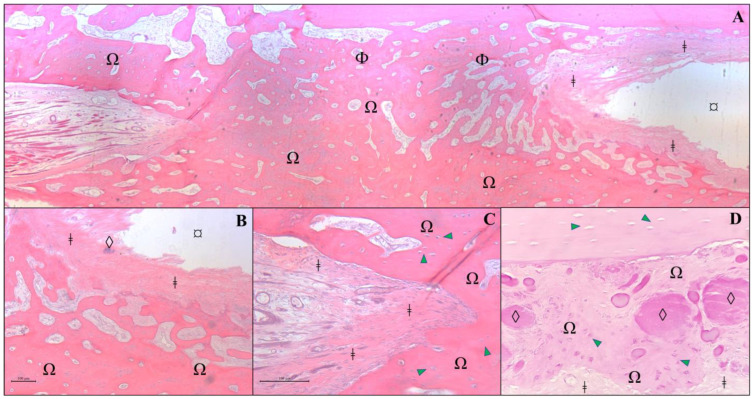
Histological analysis of a section of bone fragment/graft from an animal from Group B, 120 days after receiving an allogenous cortical bone graft. Protocol using hematoxylin and eosin in a decalcified bone fragment. (**A**) Photomicrography of grafted region at ×2.5 magnification. In the image, we can see a newly formed osteoid matrix (Ω) surrounded by bone lamellae (Φ), adipose tissue (¤), and fibrous connective tissue (ǂ). (**B**) At higher magnification (×10), a delicate demarcation is observed between the fibrous connective tissue (ǂ) surrounding the bone graft remnants (◊) and newly formed osteoid (Ω). (**C**) At ×20 magnification, fibrous connective tissue (ǂ) is observed to closely adhere to the newly formed osteoid (Ω), which is rich in osteocytes (green arrowhead). (**D**) Higher magnification of the allograft region (×20) reveals a moderate presence of amorphous and intensely eosinophilic residues (◊), surrounded by newly formed bone matrix/osteoid (Ω) with prominent osteocyte activity (green arrowhead), as well as fibrous connective tissue (ǂ).

In the decalcified bone section of the animal from Group B, osseointegration between the host bone and the allogeneic cortical bone graft was observed. It was practically impossible to differentiate the bone graft from the host bone, as the two were integrated, with only a few areas where remnants of the graft could still be found. The distinction could only be made by the type of bone present in the region of interest. The bone neoformation integrated with the lamellar bone of the host, joining the graft region to form a single bone piece. Osteoblasts and osteocytes are visible in various regions of the slide, and neovascularization is also observed in some areas of the graft (Figure 7).

## 4. Discussion

The present study evaluated the osseointegration of pure-phase β-TCP cylinder implants in critical segmental radial bone defects in rabbits. After 120 days, no osseointegration was observed between the β-TCP implant and the host bone in any of the animals from Group A. In contrast, in Group B, which received allogenous cortical bone grafts, successful osseointegration occurred. Allogeneic bone grafts present both osseoconductive and osseoinductive properties [3,7], whereas pure-phase ceramic implants have been reported to exhibit only osseoconductive characteristics [2,30]. Bioactive calcium phosphate (CaP) ceramics are considered excellent candidates for grafting materials in bone augmentations or replacements due to their ability to bind to bone and stimulate bone tissue formation [31]. Among these ceramics, β-TCP is one the most widely used and potent synthetic bone graft substitutes [32]. However, the β-TCP used in this study—presented as a cylindrical block in its pure phase and manufactured exclusively for this research—demonstrated ineffectiveness in terms of osseoconductive properties, contrasting with the results previously described by other authors [13,31,33,34,35].

Few studies have evaluated the use of β-TCP for filling critical segmental defects in long bones within veterinary medicine [36]. A bone defect that does not heal spontaneously is classified as a critical defect. In such cases, a tissue substitute or biomaterial is required to fill the gap or prevent non-union [37]. To confirm that the 7 mm segmental bone defect created in this study constituted a critical defect, the inclusion of Group C as a control group was essential. Animals in this group did not receive any type of filling, and radiographic monitoring over 120 days demonstrated bone growth; however, non-union was observed.

While numerous studies have demonstrated the osseoconductive and osseointegrative properties of β-TCP in granulated form [31,38,39,40,41,42], its application has been predominantly focused on alveolar filling and maxillary sinus lift procedures in dentistry [30,33,42,43,44,45]. In orthopedic applications, granulated β-TCP does not require strong, rigid, or stable biomechanical properties, as it is not typically used for critical defects in long bones [17,38,46,47]. For these reasons, we tested this implant in a cylindrical form.

Despite being a biocompatible material, the β-TCP implant used in this study demonstrated inert behavior, failing to promote or stimulate bone growth within its structure. These findings diverge from those reported by Cheng et al. [34], who evaluated β-TCP implants in femoral segmental defects in dogs, demonstrating promising osteoinductive properties over a 24-week postoperative period. Similarly, Takase et al. [35], in their study on a β-TCP scaffold implanted in a cylindrical bone defect on the lateral aspect of the distal femur in rabbits, reported a sufficient bioactivity of this biomaterial to induce new bone formation over a 12-week evaluation period. These discrepancies may be attributed to differences in bone defect models, the species studied, and the specific characteristics of the implants used in each study.

Even as a compacted implant, the β-TCP block exhibits porosity similar to other ceramic implants [38,40]. Pores measuring 50 μm are generally considered sufficient to promote osseoconduction [40]. However, one study using β-TCP implants with micropores smaller than 50 μm, ranging between 1 and 5 μm, also reported osseointegration, albeit in a granular implant format [39]. In our study, the β-TCP implant was classified as microporous, with a porosity of approximately 1.16 μm, as reported by the manufacturer after the sintering process. Since the implant was customized, our primary goal during sintering was to enhance mechanical rigidity and strength, which inherently reduced porosity. Our hypothesis was that this reduced porosity could provide a balance between mechanical stability, minimizing the risk of breakage, and biological integration by permitting the infiltration of blood vessels, cells, and growth factors necessary for new bone formation. However, this level of porosity proved insufficient to promote osseoconduction or integration with the host bone. It is likely that the reduced porosity resulting from the sintering process hindered the conduction of bone cells, differing from previous findings [39]. Moreover, the granular format used in prior studies may have played a significant role, as it likely facilitates better cell migration and vascularization between individual granules. This dynamic would not be possible with the cylindrical block implant, where the compact structure may act as a barrier, restricting cellular penetration and integration.

Macroscopic and radiographic evaluations revealed a difference in the timing of osteoconduction and bone integration between the two materials used in this study. The 120-day period was insufficient for the integration and remodeling of the ceramic implant. In contrast, in Group B, which received allografts, radiographic evidence of bone consolidation was observed in some animals as early as 60 days post-surgery, aligning with findings from previous studies [20,21].

Considering the resorbable properties of β-TCP bioceramic, a significant resorption of β-TCP particles and concomitant bone neoformation were expected within 3–6 months post-implantation, allowing for new trabecular bone remodeling during this period [40,42,44]. However, this was not observed in the present study, as no resorption of the biomaterial was evident 120 days post-surgery. The histological analysis revealed that all biological activity occurred around the implant, but not within it. It was expected that some degree of resorption would have occurred by the end of the evaluation period, as previously documented [3,17]. A longer observation period might have allowed for the detection of greater implant resorption and changes in its structural surface characteristics, particularly through macroscopic analysis, radiographs, and CT scans.

From a biomechanical perspective, β-TCP is a porous implant with poor biomechanical characteristics, making it susceptible to fracture under load application [48]. Despite its cylindrical and compacted block form, it likely cannot endure excessive or repetitive loads without fracturing. Consequently, prolonging the evaluation period without implant resorption and subsequent replacement by new bone could expose the implant to increased stress and fatigue, potentially leading to fracture [49]. To avoid implant fracture and mechanical interference, we aimed to isolate the biomaterial within the bone defect by performing a bridge osteosynthesis with plates and screws, effectively creating a “bypass” over the ceramic [19]. This approach successfully reduced the load and mechanical stress on the implant.

A ceramic with high porosity and low density provides an enhanced surface area for neovascularization and bone growth [2,13]. In this context, the findings of this study indicate that β-TCP did not exhibit one of the key characteristics of implants typically used as bone substitutes, which is bioactivity [50]. These findings do not align with those reported by other authors who studied β-TCP in animal models [34,35]. For osseointegration to occur, a direct connection between the implant and living bone tissue is required. This connection is facilitated by the presence of calcium and phosphorus ions in the implant, which form a chemical bond with the surrounding bone [51]. However, in this study, such integration was not observed.

Nevertheless, the β-TCP implant demonstrated good biocompatibility across all assessments performed in this study [49]. In agreement with previous radiographic studies, no tissue reactions indicative of osteomyelitis, bone resorption, or sequestration were observed [22,52]. Additionally, no fistulas or purulent drainage were present on or beneath the skin or within the surrounding tissues at the time of bone collection for histological examination. This behavior was similar to that observed in Group B, which received the bone graft. A potential concern was the occurrence of a foreign body reaction at the ostectomy site due to the implant; yet, no such reaction was observed in any case. These findings further confirm the biocompatibility of synthetic β-TCP ceramic, as previously reported [16].

During clinical evaluation, no signs of edema, local redness, swelling, pain on palpation, fistula, or abscess development were observed in any of the animals in all study groups. Even after euthanasia, when the surgical site was opened and the implanted area exposed, there was no evidence of contamination in the ceramic implant, bone graft, titanium plate, screws, or the surrounding bone and adjacent soft tissues. This was further supported by the negative culture results. These findings demonstrate that, from a biocompatibility standpoint, β-TCP is a material that could be safely used in veterinary clinical practice [34,35]. Even if it remains in the body for extended periods, it is unlikely to cause adverse reactions requiring removal.

A potential limitation of this study was the small sample size in each group, as well as the absence of treatment comparisons using β-TCP implants with different shapes and porosities. Nonetheless, our results provide valuable insights that can serve as a foundation for future investigations. Although no osseoconduction or osseointegration was observed in critical segmental radial bone defects in rabbits using a pure-phase β-TCP cylindrical implant, important data were gathered regarding the use of this biomaterial in critical bone defects. This opens new perspectives for future research exploring the application of β-TCP as a bone substitute for this type of injury.

## 5. Conclusions

In conclusion, no osseoconduction process occurred with the β-TCP biomaterial that would lead to osseointegration between the implant and the bone. The ceramic implant showed no signs of resorption, nor did it cause any inflammatory, infectious, or foreign body reactions during the 120-day period it was implanted in the critical segmental bone defect. However, the implant did not exhibit bioactive properties with the design and porosity used in this study. Further research is needed to optimize the porosity and shape of the implant in order to achieve the desired outcomes, enabling it to serve as a reliable bone substitute for cases of significant bone loss and to effectively stimulate cell adhesion between the host cells and the implant.

## Data Availability

Data are contained within the article.

## Supplementary Materials

The following supporting information can be downloaded at: https://www.mdpi.com/article/10.3390/vetsci12030200/s1, Table S1: Clinical signs evaluated after radial ostectomy and placement of a synthetic β-TCP ceramic implant or allogeneic cortical bone graft in the segmental bone defect (Groups A and B, respectively), and after radial ostectomy without defect filling (Group C).

## Abbreviations

The following abbreviations are used in this manuscript:

CaP	Calcium phosphate
β-TCP	Beta-tricalcium phosphate
µCT	Micro-computed tomography
LD	Linear dichroism
PO	Postoperative period
BMP’s	Bone morphogenetic proteins
HA	Hydroxyapatite
NDF	Without filling the defect
Cr-Ca	Craniocaudal
M-L	Mid-lateral
KVP	Kilovoltage
ROI	Region of interest
EDTA	Ethylenediamine tetraacetic acid
