# Supplementary material for: Assessment of the Osseointegration of Pure-Phase β-Tricalcium Phosphate (β-TCP) Ceramic Cylinder Implants in Critical Segmental Radial Bone Defects in Rabbits
Source: Vet Sci. 2025 Feb 26;12(3):200. doi: 10.3390/vetsci12030200 (PMC11946808; doi:10.3390/vetsci12030200)
Supplement: Supplementary file 1 [file vetsci-12-00200-s001.zip › vetsci-3456816-supplementary.pdf]

**Table S1.** Clinical signs evaluated after radial ostectomy and placement of a synthetic β-TCP ceramic implant or allogeneic cortical bone graft in the segmental bone defect (Groups A and B, respectively), and after radial ostectomy without defect filling (Group C).

| | Limb alignment | Limb support | Lameness | Surgical wound | Pain | Edema |
|---|---|---|---|---|---|---|
| **Group A (β – TCP)** | | | | | | |
| Animal A1 | Great | Great | No | SRA | + | No |
| Animal A2 | Great | Great | No | Normal | - | No |
| Animal A3 | Great | Great | No | Normal | + | No |
| Animal A4 | Great | Great | No | SRA | - | No |
| Animal A5 | Great | Great | No | Normal | - | No |
| Animal A6 | Great | Great | No | Normal | - | No |
| **Group B (Graft)** | | | | | | |
| Animal B1* | Great | Great | No | Normal | - | No |
| Animal B2 | Great | Great | No | Normal | + | No |
| Animal B3 | Great | Great | No | SRA | - | No |
| Animal B4 | Great | Great | No | Normal | - | No |
| Animal B5 | Great | Great | No | SRA | - | No |
| Animal B6 | Great | Great | No | SRA | + | No |
| **Group C (Control; no defect filling)** | | | | | | |
| Animal C1 | Great | Great | No | Normal | + | No |
| Animal C2 | Great | Great | No | SRA | - | No |
| Animal C3 | Great | Great | No | Normal | - | No |
| Animal C4 | Great | Great | No | SRA | + | No |
| Animal C5 | Great | Great | No | Normal | - | No |
| Animal C6 | Great | Great | No | Normal | + | No |

β – TCP: Beta-tricalcium-phosphate; SRA: stiches removed by animal; +: mild post-operatory pain; -: no pain; *animal died after 76 days of PO, between M3 e M4, of unknown cause.
